# The Effect of Comedy Therapy on Anxiety and Vital Signs in Liver Transplant Recipients during the Preoperative Period: A Randomized Controlled Trial

**DOI:** 10.1002/brb3.71582

**Published:** 2026-07-09

**Authors:** Hasan Sarıtaş, Şerafettin Okutan, Semra Bülbüloğlu, Serdar Sarıtaş, Dürdane Yılmaz Güven

**Affiliations:** ^1^ Division of Surgical Nursing, Department of Nursing, Faculty of Health Sciences Siirt University Siirt Turkey; ^2^ Division of Surgical Nursing, Department of Nursing, Faculty of Health Sciences Bitlis Eren University Bitlis Turkey; ^3^ Division of Surgical Nursing, Department of Nursing, Faculty of Health Sciences Istanbul Aydın University Istanbul Turkey; ^4^ Department of Medical Biology, Faculty of Medicine Malatya Turgut Özal University Malatya Turkey; ^5^ Division of Surgical Nursing, Department of Nursing, Faculty of Health Sciences Karabük University Karabük Turkey

**Keywords:** comedy therapy, liver transplantation, preoperative anxiety, vital signs

## Abstract

**Background:**

Patients awaiting liver transplantation often experience preoperative anxiety, which may negatively influence vital signs and physiological stability. Brief, low‐cost non‐pharmacological interventions such as comedy therapy may help reduce anxiety and improve selected physiological parameters. This study aimed to evaluate the effect of comedy therapy on preoperative anxiety and vital signs in liver transplant recipients.

**Methods:**

This randomized controlled trial was conducted between April 2024 and May 2025 at a liver transplant institute in Turkey. A total of 120 participants were randomly assigned to an intervention group (*n* = 60) or a control group (*n* = 60). During the preoperative period, the intervention group watched 10‐min clips from classic Turkish comedy films, while the control group received only routine care. State‐trait anxiety inventory (STAI) scores and vital signs (systolic/diastolic blood pressure, heart rate, respiratory rate, and oxygen saturation) were measured before and after the intervention. Data were analyzed using SPSS 26 with *p* < 0.05 considered significant.

**Results:**

In the intervention group, STAI scores decreased significantly after comedy therapy (*p* = 0.001), whereas no significant change occurred in the control group (*p* = 0.974). Mean pulse, systolic and diastolic blood pressure, and respiratory rate decreased, and oxygen saturation increased significantly in the intervention group (*p* < 0.05). In the control group, only a limited reduction in blood pressure was noted. Between‐group comparisons revealed significant differences in respiratory rate and oxygen saturation (*p* < 0.05).

**Conclusion:**

Comedy therapy effectively reduces preoperative anxiety and improves selected physiological parameters in liver transplant recipients. Integrating such low‐cost, non‐pharmacological interventions into surgical preparation may enhance psychological well‐being and promote physiological stability.

## Graphical abstract

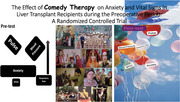

## 1 Introduction

Liver transplantation is a life‐saving treatment option for end‐stage liver failure and certain malignant diseases (Demir and Saritaş 2022). However, this complex surgical procedure causes significant stress and anxiety in patients, not only physiologically but also psychologically. Especially during the preoperative period, fear of death, uncertainty about the organ waiting process, and concerns about surgery create intense psychological pressure on patients (López‐Lazcano et al. 2021; Paglione et al. 2019). Recent studies have shown that a significant proportion of patients awaiting liver transplantation experience moderate to high levels of anxiety and depressive symptoms (López‐Lazcano et al. 2021; Paglione et al. 2019; Santos et al. 2010).

Anxiety is not limited to a mental state; it can also lead to alterations in key physiological indicators, including arterial pressure, pulse rate, and breathing frequency. (Ziyadidegan et al. 2022). It has been reported that preoperative anxiety can indirectly complicate the surgical process by significantly affecting these physiological parameters. Indeed, studies have shown that increased anxiety levels are associated with elevations in indicators such as blood pressure and heart rate, which can complicate anesthesia management and negatively affect the postoperative recovery process (AhmetovicDjug et al. 2017; Putri Nabillah et al. 2023).

Therefore, psychosocial interventions applied during the preoperative period can help balance patients' mental state and contribute to increased surgical success. In recent years, comedy therapy (laughter therapy), which is among complementary and alternative therapies, has come to the fore with its remarkable results. Comedy therapy aims to evoke positive emotions in individuals and reduce the physiological effects of stress through humorous visuals, videos, or live performances (Micozzi 2018). In a randomized controlled study conducted by Genç and Sarıtaş, comedy videos administered to surgical oncology patients were reported to cause a significant decrease in anxiety levels and stabilize blood pressure (Genç and Saritas 2020

A systematic review examining humor‐based interventions for comedy therapy (e.g., comedy videos, clown shows, and joke telling) reported that these applications have positive effects not only on psychological parameters but also on parameters related to the immune system. Significant improvements were observed in biomarkers such as natural killer (NK) cell activity, cytokine levels, immunoglobulin A (IgA), and alpha‐amylase in saliva (Hasanah et al. 2023). However, studies investigating the effects of such therapies in high‐risk patient groups, such as liver transplant recipients, are limited in number.

Yet, anxiety management may play a decisive role in the postoperative recovery process in individuals awaiting liver transplantation (Annema et al. 2018). In this context, the hypothesis that comedy therapy administered in the preoperative period may both support psychological well‐being and increase physiological stability has gained importance.

This randomized controlled study aims to investigate the effects of comedy therapy administered during the preoperative period on anxiety levels and quality of life in liver transplant recipients.

- **H_01_**: There is no significant difference in anxiety levels between liver transplant recipients who underwent comedy therapy during the preoperative period and those who did not.
- **H_02_**: There is no significant difference in vital signs (systolic‐diastolic blood pressure, heart rate, respiratory rate, oxygen saturation) between liver transplant recipients who underwent comedy therapy during the preoperative period and those who did not.
- **H_11_**: Comedy therapy administered to liver transplant recipients during the preoperative period significantly reduces anxiety levels compared to those who do not receive it.
- **H_12_**: Comedy therapy administered to liver transplant recipients during the preoperative period provides significant improvements in vital signs (systolic and diastolic blood pressure, heart rate, respiratory rate, oxygen saturation) compared to those who do not receive it.

## 2 Materials and Methods

### 2.1 Research Design and Sample

This research was structured as a randomized controlled study with a pretest–posttest format to assess the impact of Turkish comedy videos on the anxiety levels and vital signs of liver transplant recipients during the preoperative period.

The research was carried out between April 2024 and May 2025 at a specialized liver transplantation center in Turkey, the Liver Transplantation Institute (LTI). On average, this center performs approximately 270 liver transplants annually. The study sample consisted of 150 patients who were scheduled for liver transplantation at the LTI within the specified period.

The required sample size was calculated using the G*Power software (version 3.1.9.7). According to the power analysis, two groups of 60 participants each (treatment and control) were needed to achieve a statistical power of 95%, with an assumed effect size of 0.70, a significance level of 0.05, and a 95% confidence interval.

**FIGURE 1:**
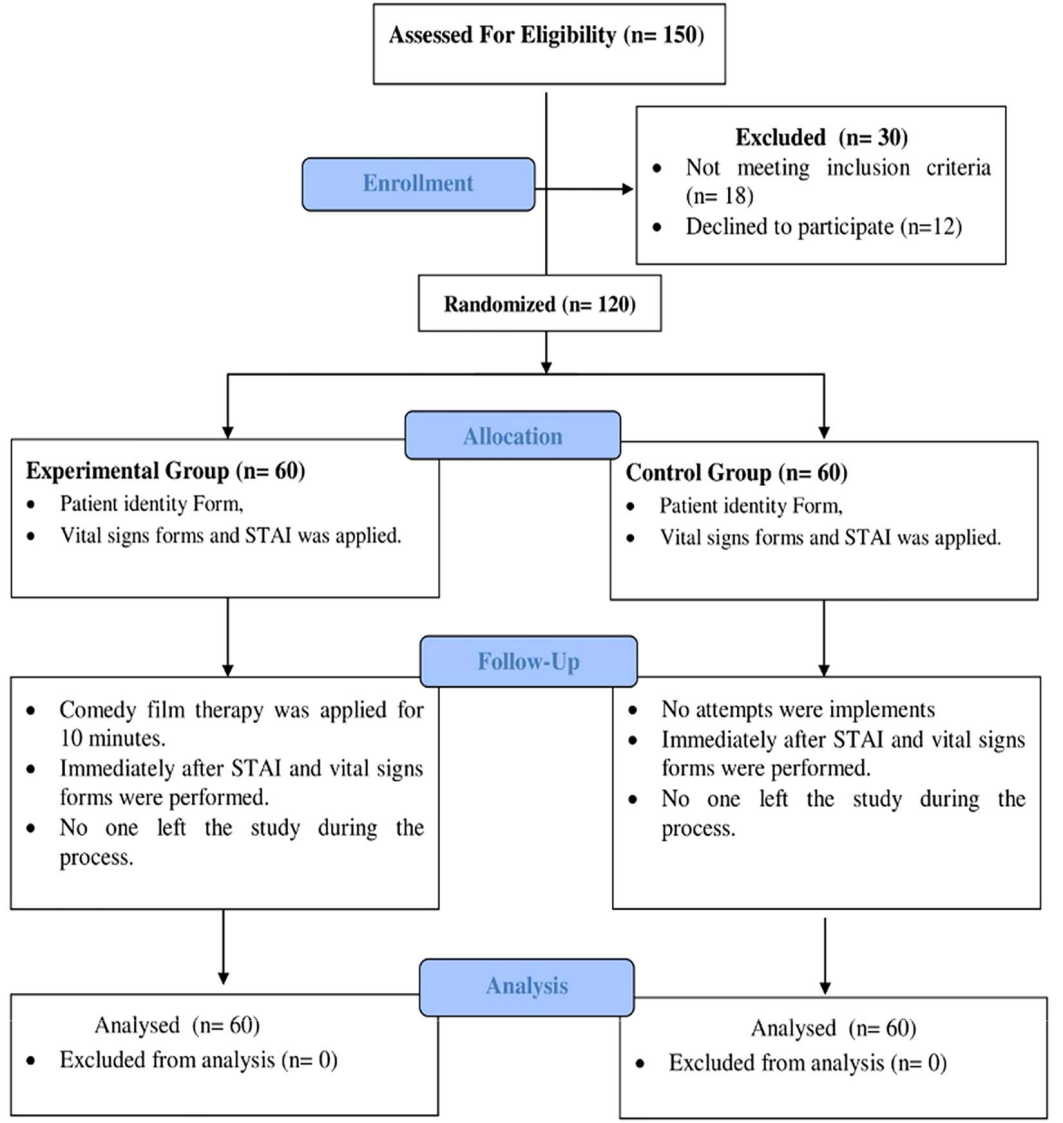
Research flow chart.

The research adhered to the guidelines outlined by the Consolidated Standards of Reporting Trials (CONSORT) (Moher et al. 2012). Participants were selected through a non‐probability random sampling approach, limited to individuals who met the inclusion criteria. A total of 18 patients who did not fulfill the eligibility requirements and 12 individuals who declined to participate were excluded from the study (see Figure 1).

Eligible participants, numbered from 1 to 120, were randomly assigned to either the intervention group or the control group in a 1:1 allocation ratio using a computer‐generated randomization sequence created with Research Randomizer software (Version 4.0). A simple randomization method was used, and no blocking or stratification procedures were applied.

### 2.2 Inclusion and Exclusion Criteria for Participants

The inclusion criteria for this study were as follows: (i) being a liver transplant candidate who voluntarily agreed to participate in the study and provided informed consent, (ii) being 18 years of age or older, (iii) being hospitalized within 24–72 h prior to the scheduled surgery, and (iv) being conscious, oriented, and able to communicate effectively.

Exclusion criteria included the following conditions: (i) a diagnosed psychiatric disorder (such as schizophrenia, major depressive disorder, or bipolar disorder), (ii) sensory or cognitive impairments (e.g., visual, auditory, or intellectual), (iii) altered consciousness due to sedation, analgesia, or the administration of potent analgesics, (iv) scheduled for an urgent/emergency liver transplant procedure, and (v) refusal to participate in the study.

### 2.3 Patient Information Form

The patient information form was constructed by the research team based on a review of current literature (Genç and Saritas 2020; López‐Lazcano et al. 2021; Paglione et al. 2019). This form aimed to gather sociodemographic and clinical data related to liver transplant candidates in the preoperative phase. It included items assessing variables such as age, sex, marital status, and education level, along with transplantation‐specific factors including etiology and donor type (living or cadaveric).

### 2.4 State‐Trait Anxiety Inventory (STAI)

The STAI is a self‐report instrument developed by Spielberger to measure individuals’ anxiety levels both in the moment (state anxiety) and as a general tendency (trait anxiety). The inventory contains 20 items rated on a four‐point Likert scale.

The Turkish adaptation and psychometric evaluation of the STAI, including its validity and reliability, were conducted by Öner and Le Compte in 1985 (Öner N 1985). In this study, the reliability of the inventory was assessed using Cronbach's alpha coefficient, which ranged between 0.83 and 0.92.

The items in the scale are rated on a 4‐point Likert scale ranging from *never* (1) to *always* (4). While some items are directly scored, others are reverse‐coded. The reverse‐scored items include statements 1, 2, 5, 8, 10, 11, 15, 16, 19, and 20. During the scoring process, reverse‐coded statements are recoded accordingly, and the total score is calculated by summing the scores of all items. The total score can range from 20 to 80, with higher scores reflecting greater levels of anxiety, whereas lower scores indicate reduced anxiety levels.

In the current study, the internal consistency of the State Anxiety subscale was found to be high, with a Cronbach's alpha coefficient of 0.931. This finding confirms the high reliability of the measurement tool employed.

### 2.5 Vital Signs Recording Form

In this study, participants’ physiological responses prior to surgery were evaluated using a “Vital Signs Recording Form.” This form was designed by the researchers, drawing upon relevant literature, to monitor and document the vital signs of liver transplant candidates in the preoperative period (Alcântara et al. 2016; Genç and Saritas 2020). The form included key hemodynamic indicators such as pulse rate, systolic and diastolic blood pressure, respiratory rate, and peripheral oxygen saturation (SpO_2_).

### 2.6 Randomization and Data Collection

Eligible participants were randomly assigned to the intervention or control group in a 1:1 allocation ratio using a computer‐generated randomization sequence created with Research Randomizer software (Version 4.0) (G.C. Urbaniak SP. n.d). A simple randomization method was used, and no stratification or blocking procedures were applied.

The randomization sequence was generated prior to participant enrollment by an independent researcher who was not involved in data collection or outcome assessment. The allocation sequence was not accessible to the researcher responsible for participant enrollment prior to assignment. Group assignments were implemented sequentially according to the predefined randomization list at the time of participant inclusion.

Allocation concealment was not feasible due to the nature of the study setting; however, the use of a pre‐generated randomization sequence, restricted access to the allocation list, and sequential assignment were employed to minimize potential selection bias. Due to the nature of the intervention, blinding of participants and researchers was not feasible. However, outcome assessments were conducted by the same researcher using standardized procedures to ensure consistency.

Data collection was performed through face‐to‐face interviews conducted between August 2024 and February 2025 at the LTI. Since liver transplant operations at the center are typically performed between 11:00 a.m. and 2:00 p.m., data collection for this study was completed between 8:00 a.m. and 9:00 a.m. No adverse events occurred during the data collection period.

### 2.7 Intervention Group

Patients in the intervention group were first administered a patient introduction form and the STAI Form during the preoperative period, and their vital signs (systolic/diastolic blood pressure, heart rate, respiratory rate, and oxygen saturation) were recorded and evaluated.

Then, a 10‐min video compiled from selected scenes of classic Turkish comedy films, specially prepared by the researchers, was shown to the patients. The intervention consisted of a standardized 10‐min video, and the content was predetermined prior to the study and was identical for all participants in the intervention group. The selection of video content was based on its humorous nature, cultural familiarity, and appropriateness for the patient population, while avoiding emotionally distressing or inappropriate scenes.

All participants watched the video under the same conditions, using the same device and duration, to ensure consistency of the intervention. During the application, a quiet, calm, and distraction‐free environment was provided in the patient's room; devices such as monitors and phones were set to silent mode, except for the television.

Immediately after the intervention was completed, the patients' vital signs were measured again, and their anxiety levels were re‐evaluated using the STAI Form. No waiting period was applied, and all measurements were conducted immediately and consistently across participants. This standardized timing was applied to all participants to ensure comparability and minimize measurement variability. Patient privacy was protected throughout all procedures, and all measurements were conducted by the same researcher using standardized procedures.

### 2.8 Control Group

Patients in the control group were also administered the patient information form and the STAI (Form X‐1) between 8:00 a.m. and 9:00 a.m. on the day of transplantation during the preoperative period, and their vital signs were recorded.

No interventions other than routine clinical practices were performed. Routine clinical practices included standard preoperative nursing care and monitoring procedures, and no additional psychological, pharmacological, or non‐pharmacological interventions specifically targeting anxiety were applied.

After a fixed waiting period of 10 min, corresponding to the duration of the intervention in the experimental group, the patients' vital signs were measured again, and their anxiety levels were reassessed using the same questionnaire form. The waiting period and measurement procedures were standardized and applied consistently to all participants to ensure comparability between groups.

All measurements were conducted under the same conditions and using the same methods as in the intervention group, and all outcome assessments were performed by the same researcher using standardized procedures. No sedative premedication or anxiolytic medication was administered to participants during the data collection period.

### 2.9 Data Analysis

Statistical analyses were performed by an independent researcher who was not involved in the randomization or data collection processes, in order to minimize potential bias.

The data were analyzed using IBM SPSS Statistics Standard Concurrent User V 26 (IBM Corp., Armonk, New York, USA). Descriptive statistics were presented as the number of units (*n*), percentage (%), and mean ± standard deviation (x ® ± SD) values. The normality of the distribution of numerical variables was assessed using the Shapiro–Wilk normality test. The homogeneity of individual characteristics between the experimental and control groups was assessed using the chi‐square test and the Independent Samples *t*‐test, as appropriate. The Cronbach's Alpha coefficient was considered in determining the validity and reliability levels of the scales.

### 2.10 Ethical Principles

Prior to the commencement of the study, ethical approval was obtained from the Siirt University Ethics Committee (Decision Date: June 28, 2024; Session No: 176; Decision No: 2024/06/01/05). Institutional permission to conduct the study at the Liver Transplant Institute was obtained from the İnönü University Liver Transplant Institute Directorate (Permission Date: June 14, 2024; Document No: E‐93629378‐605‐453879). The study was registered in a clinical trials database (Registration No: NCT06919588).

All participants took part in the study voluntarily. Before participation, the aim, scope, and procedures of the research were clearly explained by the investigators. Written informed consent was obtained from each participant who agreed to be included. Participants were assured that their personal data would be treated with strict confidentiality, that all information collected would be used solely for scientific purposes, and that they retained the right to withdraw from the study at any time without needing to provide justification.

This randomized controlled trial was conducted and reported in accordance with the CONSORT 2010 guidelines (Moher et al. 2012).

This study was conducted in alignment with the ethical standards outlined in the Declaration of Helsinki.

## 3 Results

Table 1 summarizes the findings related to the personal characteristics of liver transplant recipients included in the study and the homogeneity analysis performed between the control and intervention (experimental) groups. When the groups were assessed across sociodemographic variables such as age, gender, marital status, employment status, and economic background, no statistically meaningful differences were identified (*p* > 0.05).

**TABLE 1:** Individual characteristics and homogeneity tests of liver transplant recipients (*n* = 120).

	Control		Intervention		Homogeneity test and Sig.
	($\bar{x}$ + SD)		($\bar{x}$ + SD)		
**Age**	48.30 (13.53)		47.0 (13.41)		*t* = 0.529, *p* = 0.598
	***n***	**%**	***n***	**%**	
**Gender**					
Female	28	46.67	27	45.0	*χ* ^2^ = 0.034 *p* = 0.855
Male	32	53.33	33	55.0	
**Marital status**					
Married	41	68.3	43	71.7	*χ* ^2^ = 0.159 *p* = 0.690
Single	19	31.7	17	28.3	
**Educational status**					
Illiterate	6	10.0	9	15.0	*χ* ^2^ = 3.652 *p* = 0.455
Literate	5	8.33	6	10.0	
Primary education	14	23.33	18	30.0	
High school	18	30.0	18	30.0	
University and above	17	28.34	9	15.0	
**Employment status**					
Government employee	9	15.0	6	10.0	*χ* ^2^ = 1.118 *p* = 0.891
Worker	8	13.33	9	15.0	
Self‐employed	7	11.67	6	10.0	
Unemployed	21	35.0	25	41.67	
Retired	15	25.0	14	23.33	
**Economic status**					
Income less than expenses	37	61.67	32	53.33	*χ* ^2^ = 4.917 *p* = 0.086
Income equal to expenses	14	23.33	24	40.0	
Income greater than expenses	9	15.0	4	6.67	
**Donor type**					
Living	57	95.0	57	95.0	– –
Cadaver	3	5.0	3	5.0	
**Liver transplant etiology**					
HBV	29	48.3	31	51.7	*χ* ^2^ = 2.743 *p* = 0.987
Alcohol‐related liver failure	5	8.3	4	6.7	
HCC	4	6.7	5	8.3	
HCV	3	5.0	1	1.7	
Budd Charia	3	5.0	2	3.3	
Wilson's hepatitis	3	5.0	3	5.0	
Autoimmune hepatitis	2	3.3	1	1.7	
Fulminant hepatitis	5	8.3	4	6.7	
Toxic hepatitis	2	3.3	4	6.7	
Cryptogenic	3	5.0	4	6.7	
Hemochromatosis	1	1.7	1	1.7


*Note*: *χ* ^2^ = chi‐square test, *t* = **independent samples *t*‐test**.

It was observed that 95% of all liver transplants were conducted using grafts from living donors, while 5% involved cadaveric donors. Analysis showed no significant variation between the groups with respect to donor source. Similarly, no statistically relevant difference was found between the groups in terms of transplant etiology. The most frequently observed cause in both groups was hepatitis B virus (HBV).

These results suggest that the experimental and control groups demonstrated comparable distributions in terms of demographic and clinical characteristics. Therefore, the sample was considered appropriate for evaluating the impact of the intervention.

Table 2 presents the pretest and posttest measurement values of preoperative vital signs in both the experimental and control groups, composed of patients awaiting liver transplantation. It also includes intra‐group and inter‐group statistical comparisons for each variable.

**TABLE 2:** Comparison of preoperative vital signs of experimental and control groups based on pre‐test and post‐test values (*n* = 120).

	Experimental group	Control group	Comparison of two groups
	$\bar{x}$ ± Sd (*n* = 60)	$\bar{x}$ ± Sd (*n* = 60)	Test and Sig.
Pulse (bpm)			
Pre‐test	74.35 ± 10.17	71.833 ± 11.79	*t* = 1.251, *p* = 0.213
Post‐test	67.07 ± 12.48	71.55 ± 13.15	*t* = 1.915, p = 0.058
Intra‐group comparison	*t* = 0 4.831, *p* = 0.001**	*t* = 0.228, *p* = 0.820	
Systolic blood pressure			
Pre‐test	127.47 ± 17.69	127.26 ± 15.55	*t* = 0.066, *p* = 0.948
Post‐test	122.58 ± 10.94	125.31 ± 14.42	*t* = 1.169, *p* = 0.245
Comparison of repeated measurements	*t* = 3.734, *p* = 0.001**	*t* = 3.453, *p* = 0.001**	
Diastolic blood pressure			
Pre‐test	76.23 ± 16.04	75.50 ± 13.86	*t* = 0.268, *p* = 0.789
Post‐test	71.68 ± 8.92	73.88 ± 13.04	*t* = 1.079, *p* = 0.283
Comparison of repeated measurements	*t* = 3.750, *p* = 0.001**	*t* = 2.179, *p* = 0.033*	
Respiratory rate (rpm)			
Pre‐test	13.62 ± 2.23	14.48 ± 2.37	*t* = 2.059, *p* = 0.042*
Post‐test	13.05 ± 1.35	14.20 ± 2.23	*t* = 3.419, *p* = 0.001**
Comparison of repeated measurements	*t* = 2.312, *p* = 0 0.024*	*t* = 1.737, *p* = 0.088	
O2 saturation (%)			
Pre‐test	96.42 ± 1.24	95.61 ± 2.16	*t* = 2.485, *p* = 0.014*
Post‐test	97.28 ± 1.11	95.71 ± 1.90	*t* = 5.508, *p* = 0.001**
Comparison of repeated measurements	*t* = −5.595, *p* = 0.001**	*t* = −0.609, *p* = 0.545	

*Note*: t: *t*‐test in dependent groups.

^*^ *p* < 0.05, ^**^ *p* < 0.001.

Before the intervention (comedy therapy), there were no statistically significant differences between the groups in terms of mean pulse, systolic blood pressure, or diastolic blood pressure (*p* > 0.05). However, significant differences were observed in the mean respiratory rate and oxygen saturation (%) values (*p* < 0.05). Although statistically significant differences were observed in baseline respiratory rate and oxygen saturation, the magnitude of these differences was small and remained within normal physiological ranges; therefore, they are unlikely to be clinically meaningful.

After the comedy therapy session (posttest), no statistically significant differences were identified between the groups with regard to mean pulse, systolic blood pressure, or diastolic blood pressure (*p* > 0.05). In contrast, the experimental group showed a lower respiratory rate and a higher oxygen saturation (%) compared to the control group, and these changes were statistically significant (*p* < 0.05).

Within‐group comparisons revealed that participants in the experimental group experienced significant reductions in pulse, systolic blood pressure, diastolic blood pressure, and respiratory rate from pretest to posttest (*p* < 0.05). Oxygen saturation (%) significantly increased following the intervention (*p* < 0.05).

In the control group, only a limited reduction in systolic and diastolic blood pressure values was detected, which was statistically significant (*p* < 0.05); however, no significant change was observed in heart rate, respiratory rate, or oxygen saturation (%) values between pretest and posttest (*p* > 0.05).

Table 3 presents the pretest and posttest STAI scores of the experimental and control groups, along with the results of intra‐group and inter‐group statistical analyses.

**TABLE 3:** Comparison of STAI levels of experimental and control groups according to pre‐test and post‐test.

	Experimental group	Control group	Intra‐group comparison
	$\bar{x}$ ± Sd (*n* = 60)	$\bar{x}$ ± Sd (*n* = 60)	Test and Sig.
STAI			
Pre‐test	47.85 ± 6.87	46.71 ±11.09	*t* = 0.673, *p* = 0.503
Post‐test	40.63 ± 4.61	46.73 ± 10.29	*t* = 4.189, *p* = 0.001^**^
Intra‐group comparison	*t* = 9.378, *p* = 0.001**	*t* = −0.033, *p* = 0.974	

*Note*: t: *t*‐test in dependent groups.

^*^ *p* < 0.05, ^**^ *p* < 0.001.

Before the intervention (pretest), no statistically significant difference was observed between the experimental and control groups in terms of mean STAI scores (*p* = 0.503). However, following the comedy therapy (posttest), a statistically significant difference emerged between the groups. Specifically, the experimental group exhibited a significant reduction in mean STAI score, while the control group's score remained stable. This between‐group difference was statistically significant (*p* = 0.001).

In the within‐group comparison, the decline in STAI mean scores from pretest to posttest in the experimental group reached statistical significance (*p* = 0.001). In contrast, no significant change was detected in the control group, and this result was not statistically significant (*p* = 0.974).

## 4 Discussion

Comedy and humor are considered effective, safe, and easy‐to‐implement interventions for reducing anxiety and stress. The literature reports that such interventions have beneficial effects on vital signs—particularly blood pressure—and contribute to psychological relief and overall well‐being (Genç and Saritas 2020; Sarink and García‐Montes 2023; Zhao et al. 2020). Similarly, in our study, a significant decrease in heart rate and mean systolic and diastolic blood pressure was observed in the experimental group who watched comedy films (Table 2).

When examining studies related to the preoperative period, Genc and Sarıtaş’s randomized controlled study also reported that watching comedy videos before surgery reduced anxiety levels and blood pressure values in surgical oncology patients (Genç and Saritas 2020). The calming effect of comedy and humor applications on the cardiovascular system is being examined in a multidimensional manner in current research. Humor and laughter improve vascular function, reduce stress‐related hormone levels, and positively affect cardiovascular health by increasing overall well‐being (Saffi et al. 2023; Sugawara et al. 2010). These effects are more pronounced in individuals recovering from cardiovascular diseases or living with chronic conditions such as hypertension (Susanti et al. 2022). Our findings align with other studies indicating that humor may contribute to reductions in blood pressure.

In the present study, a statistically significant difference was identified in average respiratory rates between the experimental and control groups after the comedy‐based intervention. Following the intervention, decreases in respiratory rate were observed in both the experimental and control groups. However, the decrease in respiratory rate in the experimental group was statistically significant compared to the pre‐test value. Given the baseline difference between groups, the results were interpreted based on within‐group (pretest–posttest) changes rather than absolute between‐group comparisons, thereby reducing the potential impact of baseline imbalance on the interpretation of the findings. These findings suggest that humor‐oriented interventions may exert not only psychological but also physiological benefits by influencing autonomic nervous system activity. As respiratory rate is a direct physiological indicator of stress and anxiety, a reduction in this measure reflects enhanced relaxation and engagement of the parasympathetic nervous system.

In the literature, a study conducted with children with tracheostomies found that interventions involving comical animations and puppet interactions significantly reduced respiratory rate (Valadkhani et al. 2022). Similarly, watching comedy videos has been observed to reduce anxiety levels and indirectly improve respiratory function in surgical patients(Micozzi 2018).

Although the effect of comedy‐based interventions on respiratory parameters has been reported less frequently in the literature, Genc and Sarıtaş’s study stated that there was a decrease in respiratory rate in the experimental group after video viewing in post‐test measurements, but this difference was not found to be statistically significant in group comparisons (Genç and Saritas 2020; Sarıtaş et al. 2019). On the other hand, in a study conducted by Zhao and colleagues, a humor intervention applied to nursing home residents over eight weeks resulted in positive changes not only in anxiety levels but also in physiological and behavioral responses (Zhao et al. 2020). These findings emphasize the long‐term effects of humor on the autonomic nervous system and support the idea that the decrease in respiratory rate is related to a decrease in stress levels. This study focused not only on within‐group changes but also on between‐group differences. Following the intervention, in which the experimental group watched a 10‐min compilation of classic Turkish comedy films during the preoperative period, significant improvements were observed in both STAI scores and vital signs such as respiratory rate and oxygen saturation. In particular, when compared to the control group, the experimental group exhibited a significantly lower respiratory rate and higher oxygen saturation, indicating the physiological effects of the intervention. Likewise, the reduction in STAI scores was not only significant within the experimental group but also statistically greater than that of the control group, supporting the psychological effectiveness of the intervention. These findings suggest that comedy therapy—being a short‐term, non‐invasive, and cost‐effective method—may support psychophysiological balance during surgical preparation. The use of a randomized controlled trial design and the identification of significant differences between groups enhance the causal inference and scientific strength of the study's findings.

In conclusion, humor's ability to slow breathing by reducing stress responses creates a homeostatic balancing effect and contributes to physiological stability during the surgical preparation process. Sarink and García‐Montes' systematic review also noted that humor stimulates a relaxation response parallel to a decrease in anxiety levels and that this effect can produce measurable outputs in physiological systems (Sarink and García‐Montes 2023). In this context, the significant decrease in respiratory rate observed in our study demonstrates the potential of comedy therapy to reduce preoperative physiological stress responses and is consistent with similar findings in the literature.

In our study, a significant decrease in anxiety levels was observed in patients after comedy application (Table 3). This finding is consistent with previous studies showing that watching comedy films has positive effects on anxiety in surgical patients. Various studies have shown that comedy films, which are among visual‐auditory distractions, can significantly reduce anxiety levels in individuals undergoing surgery (Chow et al. 2016; Genç and Saritas 2020; Kapıkıran et al. 2025; Ketsuwan et al. 2021; Sarıtaş et al. 2019).

Studies conducted on surgical patients, in particular, have shown that watching comedy content leads to a significant reduction in anxiety levels. For example, in a study involving oncology patients, individuals who watched comedy videos had lower state anxiety scores compared to participants who did not receive any intervention, and this finding supports the anxiolytic effect of humor(Genç and Saritas 2020; Sarıtaş et al. 2019). Similarly, patients who watched films during Extracorporeal Shock Wave Lithotripsy (ESWL) surgery experienced significantly lower anxiety levels compared to the control group (Ketsuwan et al. 2021).

The data obtained in this context support the potential of comedy‐based interventions as an effective method for providing psychological relief during the preoperative process and reducing anxiety.

## 5 Conclusion

This randomized controlled study demonstrated that comedy therapy administered during the preoperative period in liver transplant candidates significantly reduced anxiety levels and improved certain physiological indicators, particularly respiratory rate and oxygen saturation. These findings highlight the potential of comedy‐based interventions as non‐invasive, low‐cost, and easily implementable strategies to alleviate psychophysiological stress before surgery. Although the effects were measured only in the immediate preoperative period, the between‐group comparisons indicate that the intervention produced statistically meaningful outcomes beyond intra‐group changes. Future studies should explore the long‐term clinical implications of such interventions in postoperative settings.

Recommendations are as follows:

- Noninvasive and cost‐effective interventions such as comedy therapy should be considered as part of routine practices in the surgical preparation process.
- Investigating the effects of humor‐based interventions in different surgical branches and patient groups will increase the generalizability of the findings.
- Including humor among anxiety management strategies in clinical nursing practices may contribute to the quality of patient care.
- Future studies should evaluate long‐term effects and examine their relationship with physiological and psychological outcomes in the postoperative period.

## 6 Limitations of the Research

This study has several limitations. First, the intervention was limited to a single, brief (10‐min) session administered only during the preoperative period, with no postoperative follow‐up. Therefore, the long‐term effects of comedy therapy on clinical outcomes remain unknown. Second, all data were collected from a single transplant center, which may limit the generalizability of the findings. Third, individual variations in humor perception could have influenced the effectiveness of the intervention. Additionally, the absence of a placebo or attention‐control condition limits the ability to distinguish the specific effects of the intervention from nonspecific factors such as attention or distraction. Furthermore, blinding of participants and researchers was not feasible due to the nature of the intervention, which may introduce a potential risk of measurement bias, particularly for subjective outcomes such as anxiety. Lastly, external stressors such as waiting time and environmental noise could not be fully controlled and may have impacted participant responses.

## Author Contributions

**Semra Bülbüloğlu**: supervision, resources, writing – review and editing. **Hasan Sarıtaş**: conceptualization, methodology, investigation, data curation, formal analysis, writing – original draft, project administration, visualization, software. **Şerafettin Okutan**: methodology, formal analysis, validation, writing – review and editing, software. **Dürdane Yılmaz Güven**: data curation, investigation, resources, writing – review and editing. **Serdar Sarıtaş**: funding acquisition, supervision, validation, writing – review and editing.

## Ethics Statement

Ethical approval for this study was obtained from the Siirt University Ethics Committee (Decision Date: June 28, 2024; Session No: 176; Decision No: 2024/06/01/05). Institutional permission to conduct the study at the Liver Transplant Institute was obtained from the İnönü University Liver Transplant Institute Directorate (Permission Date: June 14, 2024; Document No: E‐93629378‐605‐453879). Written informed consent was obtained from all participants. The study was conducted in accordance with the ethical principles of the Declaration of Helsinki.

## Funding

The authors have nothing to report.

## Conflicts of Interest

The authors declare no conflicts of interest.

## Data Availability

The data that support the findings of this study are available from the corresponding author upon reasonable request. Due to ethical and privacy restrictions, the data are not publicly available.
